# Characterization and application of a lytic jumbo phage ZPAH34 against multidrug-resistant *Aeromonas hydrophila*

**DOI:** 10.3389/fmicb.2023.1178876

**Published:** 2023-06-21

**Authors:** Yuting Hou, Zhihao Wu, Li Ren, Yuan Chen, Yong-An Zhang, Yang Zhou

**Affiliations:** ^1^National Key Laboratory of Agricultural Microbiology, Hubei Hongshan Laboratory, Engineering Research Center of Green Development for Conventional Aquatic Biological Industry in the Yangtze River Economic Belt, Ministry of Education, Shenzhen Institute of Nutrition and Health, College of Fisheries, Huazhong Agricultural University, Wuhan, China; ^2^Guangdong Laboratory for Lingnan Modern Agriculture, Guangzhou, China; ^3^Shenzhen Branch, Guangdong Laboratory for Lingnan Modern Agriculture, Genome Analysis Laboratory of the Ministry of Agriculture, Agricultural Genomics Institute at Shenzhen, Chinese Academy of Agricultural Sciences, Shenzhen, China

**Keywords:** *Aeromonas hydrophila*, phage ZPAH34, genome, foods, biofilm

## Abstract

*Aeromonas hydrophila* is an emerging foodborne pathogen causing human gastroenteritis. *Aeromonas* species isolated from food such as seafood presented multidrug-resistance (MDR), raising serious concerns regarding food safety and public health. The use of phages to infect bacteria is a defense against drug-resistant pathogens. In this study, phage ZPAH34 isolated from the lake sample exerted lytic activity against MDR *A. hydrophila* strain ZYAH75 and inhibited the biofilm on different food-contacting surfaces. ZPAH34 has a large dsDNA genome of 234 kb which belongs to a novel jumbo phage. However, its particle size is the smallest of known jumbo phages so far. Based on phylogenetic analysis, ZPAH34 was used to establish a new genus *Chaoshanvirus*. Biological characterization revealed that ZPAH34 exhibited wide environmental tolerance, and a high rapid adsorb and reproductive capacity. Food biocontrol experiments demonstrated that ZPAH34 reduces the viable count of *A. hydrophila* on fish fillets (2.31 log) and lettuce (3.28 log) with potential bactericidal effects. This study isolated and characterized jumbo phage ZPAH34 not only enriched the understanding of phage biological entity diversity and evolution because of its minimal virion size with large genome but also was the first usage of jumbo phage in food safety to eliminate *A. hydrophila*.

## 1. Introduction

*Aeromonas hydrophila* was described as a ubiquitous ‘Jack of all trades’ in the One Health world that can colonize animals and humans, where it can trigger cutaneous or digestive infections (Lamy et al., 2021). *Aeromonas hydrophila* has been isolated from raw meat, poultry, milk, seafood, vegetables, and fish and their presence in the food environment increases the risk of human gastroenteritis, tissue infections endocarditis, and pneumonia (Awan et al., 2018). There have been several reports of foodborne outbreaks caused by *A. hydrophila*. In 2012, over 200 college students in China consumed vegetables contaminated with *A. hydrophila*, causing acute diarrhea with vomiting and fever symptoms (Zhang et al., 2012). Another outbreak of multi-drug resistant *A. hydrophila* food poisoning led to 33 cases of acute gastroenteritis in Bhutan in 2016 (Tsheten et al., 2016). Not only that, recent studies investigating the prevalence of *A. hydrophila* in various types of retail seafoods and ready-to-eat sushi presented with the high prevalence and potential virulence (Park et al., 2021). Therefore, *A. hydrophila* currently has the status of a foodborne pathogen of emerging importance.

The extensive use of antibacterial drugs in the food industry, agriculture, and aquaculture has led to an increase in drug-resistant strains, which have become a public health risk (Thayumanavan et al., 2003; Boor, 2006; Chen et al., 2021). Studies on the antibiotic resistance of *A. hydrophila* isolates revealed the existence of numerous strains that are extremely resistant to some antibiotics used in clinical practice, making it potential challenge to treat diseases brought on by *A. hydrophila* (Daskalov, 2006). The presence of multiple drug resistance phenotype and biofilm formation capacity of *Aeromonas* species in raw seafood in Bangkok, Thailand, poses a potential health hazard to humans (Santajit et al., 2022). Bacterial biofilms formed on the surfaces of biotic and abiotic, including living tissue, food, medical devices, water piping systems, glass and plastics, also contribute to bacterial resistance (Jahid et al., 2014; Du et al., 2016). The presence of biofilms and antibiotic resistance mechanisms increase the prevalence of multi-drug resistant strains, and the prevention and treatment of related diseases caused by *A. hydrophila* is a major challenge in research (Dong et al., 2021; Nithin et al., 2021). Therefore, it is particularly necessary to develop new strategies to eliminate *A. hydrophila*.

Bacteriophages (phages) are viruses that specifically kill bacteria (Salmond and Fineran, 2015). Phages are part of the microbial community and have been isolated from a variety of food products, and several researches confirmed their safety in applications (Montso et al., 2021). As effective alternative antimicrobials for controlling bacterial contamination, phages are increasingly applied in the field of food industry, and some phage products are used as approved by Food and Drug Administration (FDA) (Pinto et al., 2020; Ramos-Vivas et al., 2021). Phages can effectively reduce the bacterial load in various food matrices (Han et al., 2013; Cong et al., 2021). Moreover, phages can be applied to the decontamination of food processing facility surfaces to prevent bacterial contamination (Lewis and Hill, 2020; Federici et al., 2021). Based on the phage genome size, larger than 200 kb are classified as jumbo phages (Yuan and Gao, 2017). Jumbo phages have an arsenal of techniques to counter bacterial defense mechanisms, offering phage with a broad level of resistance (Nazir et al., 2021). Owing to their infrequent isolation and incomplete characterization, there have been no reports on the application of jumbo *Aeromonas* phages in food biocontrol (Duponchel and Fischer, 2019).

To address the challenge of multidrug-resistant *A. hydrophila* contamination in the food chain, the lytic activity, antimicrobial potential on biofilms, and biological characterization of a new lytic phage ZPAH34 have been evaluated in this study. Genomic analysis and phylogenetic analysis revealed that phage ZPAH34 belongs to jumbo phage with minimal head and tail structure, and is classified into a new genus, *Chaoshanvirus*. Further, the biocontrol capability of phages ZPAH34 was evaluated on food matrices indicating its practical potential as a novel biocontrol strategy.

## 2. Materials and methods

### 2.1. Phage isolation and purification.

*Aeromonas hydrophila* ZYAH75 (GenBank accession no. NZ_CP016990) was used for phage isolation, which is a representative MDR strain. Phages were isolated from water samples collected from the lake in Wuhan, China, according to a previous study with modification (Yan et al., 2020). These water samples were centrifuged at 10,000 × *g* for 10 min and filtered through a 0.22 μm filter membrane (Millipore, Ireland) to obtain a relatively pure solution. 5 ml of suspensions were mixed with 10 ml Luria Bertani (LB) Broth and 2.5 ml of overnight-cultured *A. hydrophila* ZYAH75, then incubated at 28°C. After centrifugation and filtration, the phage purification was performed using a double-layer agar plate method. A single plaque was selected from a plate and placed in LB that had been pre-inoculated with overnight-cultured host bacteria to purify the phages. Repeating the above steps at least three times to obtain uniformly sized phages. The purified phage was stored at 4°C.

### 2.2. Determination of phage lytic ability

The lytic efficiency of collected phages including ZPAH12, ZPAH21, ZPAH29, ZPAH34, ZPAH71, ZPAH85, ZPAH103, ZPAH106, ZPAH109, and ZPAH118 was evaluated in a 96-well microtiter plate. The experimental group comprised 100 μl of *A. hydrophila* ZYAH75 mixed with 100 μl of phage suspension at various multiplicity of infection (10^2^–10^8^ PFU/ml), and fresh LB served as the control group. The plates were incubated at 28°C on an orbital shaker at 160 rpm for 12 h and an optical density at 600 nm (OD_600_) of each well was measured every hour using a microplate reader (Infinite M200 Pro, Tecan, Switzerland).

### 2.3. Biofilm formation and degradation

With reference to Islam’s study (Islam et al., 2021), the ability of phage to inhibit and remove *A. hydrophila* biofilms was assessed, respectively, on both plastic and glass surfaces.

To detect the inhibitory effect of phage on biofilm formation, a 96-well plate (VWR, American) was inoculated with 160 μl LB medium, 20 μl overnight cultures of *A. hydrophila* ZYAH75 and 20 μl phage with a concentration of 10^8^ PFU/ml or 10^9^ PFU/ml. The microplates were incubated at 28°C for 72 h to allow bacteria to adhere and form biofilm. The control group was treated with PBS buffer. Following incubation, the supernatants were removed and each well was gently washed three times with 200 μl PBS to remove planktonic cells. Each well was fixed with 200 μl methanol for 30 min and then stained with 1% crystal violet solution for 20 min and washed three times with PBS. The stained cells were eluted with 33% glacial acetic acid. The OD_600_ in each well was measured by a microplate reader (Judith et al., 2005). To measure the biofilm inhibition on glass surface, sterile glass coverslips (Biosharp, China) were placed on the bottom of the 12-well plate (VWR, American) prior to incubation. Three hundred μl of 10^8^ PFU/ml and 10^9^ PFU/ml phages were mixed with 300 μl of *A. hydrophila* ZYAH75 and 2.4 ml of LB each well. The mixture was cultured at 28°C for 72 h to allow them to interact. Then bacterial solutions were carefully removed by pipette and phages at a concentration of 10^8^ PFU/ml or 10^9^ PFU/ml were added to mature biofilms to incubate at 28°C for 12 h. Wells were washed thrice with PBS to remove planktonic bacteria. The biofilm cells on the glass coverslips were thoroughly detach with sterile cotton swab and re-suspended with PBS. The bacterial suspension were serially diluted and 100 μl diluent was inoculated on LB plate for the viable count.

To measure the biofilm clearance effect, 20 μl *A. hydrophila* ZYAH75 were inoculated into 180 μl LB medium in each well of the 96-well microplate. The bacterial supernatants were removed from the mature biofilm after 72 h of incubation. Then 200 μl phages at a final concentration of 10^8^ PFU/ml or 10^9^ PFU/ml were added, mixed and incubated at 28°C for 12 h. The control group was treated with PBS buffer. Following incubation, the supernatants were removed and each well was gently washed three times with 200 μl PBS to remove planktonic cells. Each well was tested using crystal violet staining according to the above steps. To measure biofilm clearance capacity on glass surface, 300 μl of *A. hydrophila* ZYAH75 and 2.7 ml of LB were added to each well for 72 h to form biofilm. Then bacterial solutions were carefully removed by pipette and phages at a concentration of 10^8^ PFU/ml or 10^9^ PFU/ml were added to mature biofilms to incubate at 28°C for 12 h. Wells were washed thrice with PBS to remove planktonic bacteria. Cell counts was quantified by direct plating according to the above steps. Experiments were conducted in triplicate.

### 2.4. Morphology analysis of ZPAH34

A high concentration of phage suspension was obtained by ultracentrifugation at 30,000 × *g* for 2 h. Phage suspension with a concentration of 10 log PFU/ml was dropped on a copper grid which was allowed to stick for 5 min. The excess liquid was drained with filter paper, and 2% phosphotungstic acid was dropped on the copper grid and negatively stained for 1 min. The copper grid was transferred to a Petri dish and dried at room temperature. The phage morphology was observed by a transmission electron microscope (TEM; Hitachi H-7000FA, Japan) at 75 kV (Liu et al., 2020).

### 2.5. Adsorption rate and one-step growth curve assay

The adsorption rate experiment of phage was determined according to a modified method described by the previous description (Islam et al., 2021). The logarithmic growing *A. hydrophila* ZYAH75 was mixed with phage suspensions at the MOI of 0.001 and incubated at 28°C for 25 min. Hundred μl of mixture was taken into a centrifuge tube containing 900 μl of PBS at 5, 10, 15, 20 min and the mixture was then centrifuged at 8000 × *g* for 1 min. The titer of free phages was determined by the double-layer agar assay.

A one-step growth curve experiment was conducted following the respective method described (Zhou et al., 2020). In brief, the phage was incubated with bacterial culture (10^8^ CFU/ml) at the optimal MOI of 0.001. The mixture was cultured for 15 min at 28°C to allow the phage to adsorb on the host bacteria and then centrifuged for 8,000 × *g* for 2 min to remove unabsorbed phages. The bacterial particles were washed twice with 1 ml LB, and then transferred into 9 ml of LB incubated for 120 min at 28°C. The sample was collected in aliquots at 0, 5, 10, 15, 20 min, and every 10 min thereafter until 120 min and instantly centrifuged at 8000 × *g* for 2 min. The phage titers were tested using the double-layer agar method. The number of released phages can ascertain the eclipse and latent period (Hamdi et al., 2017). Three parallel experiments were carried out.

### 2.6. Determination of pH and temperature tolerance

For the pH tolerance test, phage suspensions (10^8^ PFU/ml) were transferred to LB with pH ranging 2–13 and then incubated at 28°C for 1 h (Liu et al., 2020). For the thermal tolerance assay, 1 ml aliquot of phage suspensions (10^8^ PFU/ml) was incubated for 30 and 60 min at a temperature range from 30°C to 80°C. The results of pH and thermal stability tests were determined by measuring the number of phage plaques using the double-layer agar method.

### 2.7. Genome sequencing of ZPAH34

Phage genomic DNA was extracted according to a previous study (Akmal et al., 2020). The phages were first treated using DNase I and RNase A (TaKaRa, Japan) to prevent bacterial nucleic acid interference (Chen et al., 2020). Whole-genome sequencing of phage ZPAH34 was performed by using an Illumina HiSeq platform, with the accurate sequence reads assembled by MicrobeTrakr plus (v0.9.1) software. Open reading frames (ORFs) of ZPAH34 were predicted using MicrobeTrakr plus (v0.9.1) software, annotated against the Non-Redundant protein databases of the NCBI using the BLASTP algorithm^1^ (Yan et al., 2020). Phage genome sequences were visualized by the CGView Server (Stothard et al., 2019). Proteins similarities between phages were analyzed using BLASTP in NCBI database. All annotated genes were detected the presence of antimicrobial resistant genes (ARGs) with the ARG-ANNOT database^2^ and the presence of potential virulence factors with the VFDB database.^3^ The major capsid protein was applied for phylogenetic analysis to determine the taxonomy of phage, and a phylogenetic tree was constructed via IQ-tree, and visualized using iTol (Minh et al., 2020). The complete genome sequence of the phage ZPAH34 has been submitted to the NCBI GenBank database (Accession Number: OM810292).

### 2.8. Application of ZPAH34 in different food

To assess the ability of phage ZPAH34 to inhibit the growth of *A. hydrophila* on food, samples of lettuce and grass carp purchased from a local supermarket were used to determine bactericidal activity, respectively. In the lettuce experiment, leaves were cut into 1 cm × 1 cm size with sterile scissors, then soaked in 75% ethanol and exposed to UV to eliminate bacterial contamination. The sterile leaves were immersed in 200 ml of bacteria suspension with a concentration of 10^6^ CFU/ml for 5 min, and then air-dried to make the bacteria uniformly adhere to the surface of the leaves. The sample was then immersed in 200 ml phage suspensions at an MOI of 10 (10^7^ PFU/ml) or 100 (10^8^ PFU/ml) for 5 min, respectively (Yang et al., 2019). The samples were treated with the same volume of PBS in the control group. The samples were randomly divided into two groups and incubated at 4 and 25°C for 6 h, respectively. Samples were collected at four time points of 0, 1, 3, and 6 h, homogenized with a grinding rod, and 15 μl of serially diluted suspension was coated on LB plates for detection of viable bacteria (Guo et al., 2021).

In the fish fillet experiment, fish muscles of grass carp were sliced into 2 cm × 1 cm thin slices with a sterile knife. The fillets were sterilized with 75% ethanol, exposed to UV and then washed with sterile water to eliminate other bacterial interference. The bacterial solution was prepared as described above. After natural air drying, the fillet samples were immersed in 200 ml of *A. hydrophila* ZYAH75 at a density of 10^6^ CFU/ml for 5 min to allow the host bacteria to attach to the fish fillet. The inoculated samples were removed, air-dried for 30 min, and transferred to 200 ml of phage suspensions at an MOI of 10 (10^7^ PFU/ml) or 100 (10^8^ PFU/ml) for 5 min, respectively. The control group used the same volume of PBS to treat the samples. The fillet samples were randomly divided into two groups that were incubated at either 4 or 25°C for 6 h. After incubation for 0, 1, 3, and 6 h, the samples were placed in a grinder with PBS buffer, homogenized and diluted, and then coated on LB plates for counting viable bacteria. The above experiments were conducted in three parallels.

### 2.9. Statistical analysis

GraphPad Prism (San Diego, CA, USA) was used for the statistical analysis of data. In the experiments on the application of food and biofilm, data are expressed as mean ± SD and significant differences between the experimental and control groups were tested by *t*-test. Data from the experimental group were considered statistically significant when the value of *p* was <0.05.

## 3. Results

### 3.1. Isolation of phages infecting *Aeromonas hydrophila* MDR strain ZYAH75

**Figure 1:**
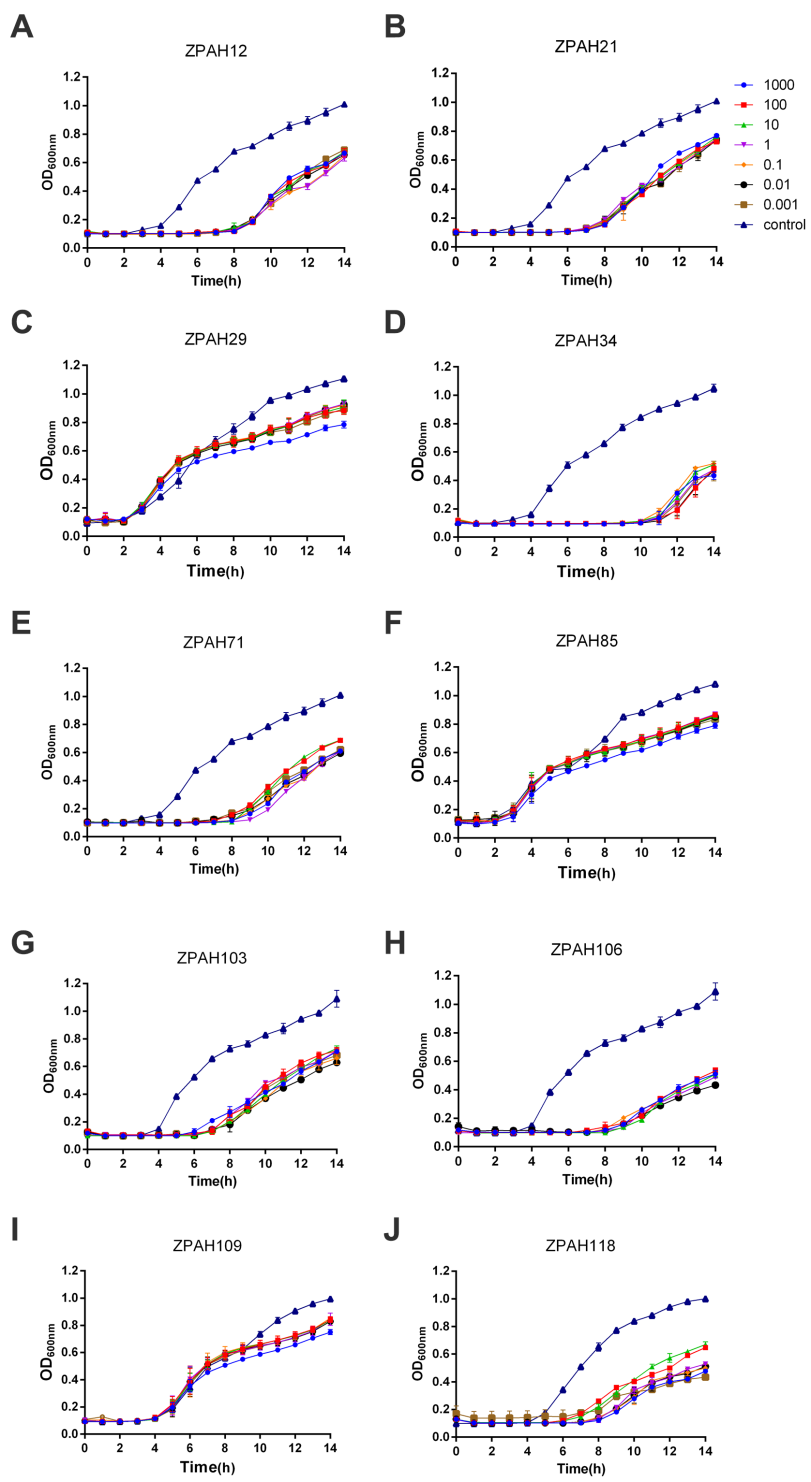
Lytic curves of phages on *Aeromonas hydrophila* ZYAH75. Lytic ability of phages ZPAH12 **(A)**, ZPAH21 **(B)**, ZPAH29 **(C)**, ZPAH34 **(D)**, ZPAH71 **(E)**, ZPAH85 **(F)**, ZPAH103 **(G)**, ZPAH106 **(H)**, ZPAH109 **(I)**, and ZPAH118 **(J)** against *A. hydrophila* ZYAH75 was determined at various range of infections (MOIs of 0.001, 0.01, 0.1, 1, 10, 100, and 1,000), respectively. The control group was added LB to the bacterial suspension.

Ten phages isolated from water samples were evaluated for lytic activity against *A. hydrophila* MDR strain ZYAH75. All phages could inhibit the growth of host bacteria to different degrees compared to the control group (Figure 1). In particular, the density of bacteria treated with phage ZPAH34 was significantly lower than the other nine phages within 12 h treatment, exhibiting remarkable inhibition at different MOIs (Figure 1D). Phage ZPAH34 with the most effective lytic ability was chosen for further study.

### 3.2. Biofilm inhibition and clearance ability of phage ZPAH34

**Figure 2:**
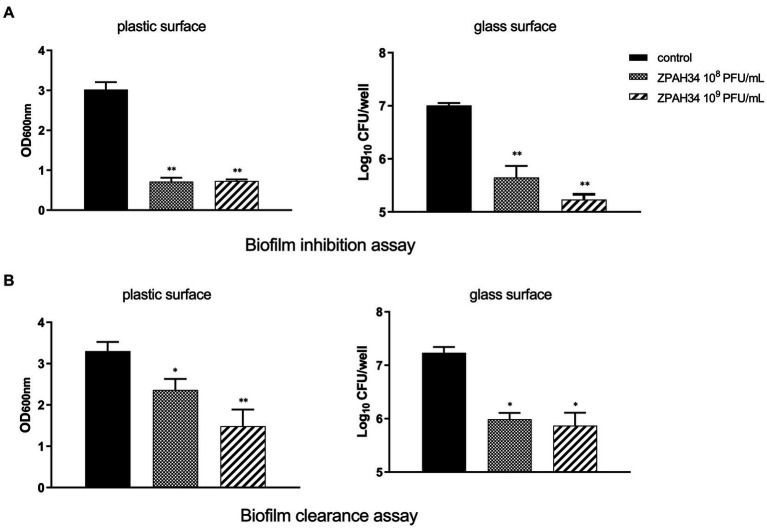
Effect of ZPAH34 on *A. hydrophila* ZYAH75 biofilms. Biofilm inhibition ability of ZPAH34 in 96-well plates and on glass coverslip **(A)** incubated with bacterial suspension at 28°C for 72 h. Clearance ability of ZPAH34 on mature biofilm in 96-well plates and on glass coverslip **(B)** after treatment for 12 h. The crystal violet staining method was used to determine the OD_600_ in each well. *p < 0.05; **p < 0.01.

The efficacy of phage ZPAH34 against *A. hydrophila* ZYAH75 biofilm on two materials of plastic and glass was assessed. The inhibition of *A. hydrophila* ZYAH75 biofilm was determined after co-incubation of host bacteria with phage. Incubation with 10^8^ PFU/ml and 10^9^ PFU/ml of phage ZPAH34 resulted in 76.35 and 75.76% reduction of the biofilm (*p* < 0.01), respectively, indicating ZPAH34 markedly inhibited the formation of biofilm (Figure 2A). Similar results were observed on glass coverslips, treatment with 10^8^ PFU/ml and 10^9^ PFU/ml of phage ZPAH34 resulted in 1.35 log CFU/ml and 1.77 log CFU/ml reduction of bacterial count, respectively (*p* < 0.05) (Figure 2A).

In the biofilm clearance assay, 28.48 and 54.48% of the matured biofilms were cleared with 10^8^ and 10^9^ PFU/ml ZPAH34, respectively, after 12 h treatment, as detected by the colorimetric method (Figure 2B). On glass coverslips, the viable bacteria count determined 1.24 log10 and 1.36 log10 CFU/ml bacterial reduction in mature biofilms incubated with 10^8^ and 10^9^ PFU/ml ZPAH34 for 12 h, respectively (*p* < 0.01) (Figure 2B), indicating ZPAH34 could not only effectively inhibit biofilm formation, but also disrupt the mature biofilm of *A. hydrophila* on the two material surfaces.

### 3.3. Genomic analysis of ZPAH34

**Figure 3:**
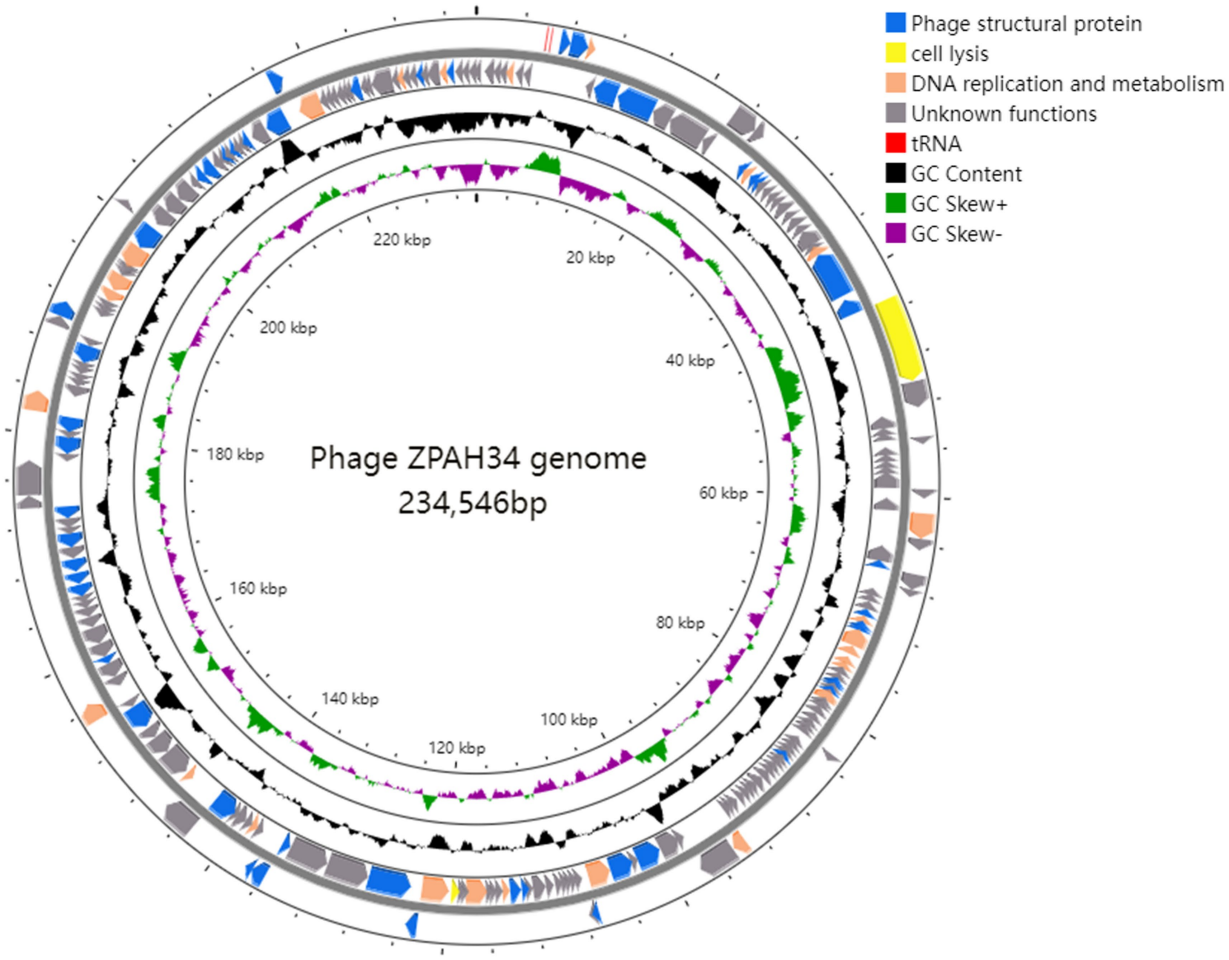
Genomic map of ZPAH34. Circles display (from the outside): (1) the gene coding regions transcribed in the clockwise or the counterclockwise direction. (2) G + C % content. (3) GC skew plot of G-C/G + C are present by green and purple. (4) The inner circle indicates the full length of the ZPAH34 genome. The open reading frames represent genes encoding structural proteins, cell lysis proteins, DNA replication and metabolism proteins, and tRNAs are denoted by the color blue, yellow, oranges, and red, respectively.

The complete genome of phage ZPAH34 was sequenced and the general features are shown (Figure 3). ZPAH34 genome consisted of 234,546 bp belongs to jumbo phage, with typical double-stranded DNA and overall GC content of 36%. A total of 234 open reading frames (ORFs) and 2 tRNAs were predicted by RAST in the phage ZPAH34 genome. All predicted proteins were annotated by NCBI BLASTP, and predicted functions of 80 ORFs were mainly involved in structural protein assembly, cell lysis and DNA replication, and metabolism, and the rest were hypothetical proteins with unknown functions. The genome of ZPAH34 encodes the proteins responsible for DNA replication and transcription such as DNA polymerases (ORFs 174, 212) and RNA polymerases (ORFs 36, 37, 192) to initiate early transcription. The endolysin (ORF 38) and putative transglycosylase (ORF 128) of ZPAH34 were identified. The gene encoding for the phage nuclear shell protein named chimallin (ORF 210) was also identified. No repressor proteins and integrases associated with lysogenic conversion were detected in the ZPAH34 genome. The absence of antibiotic resistance genes and virulence-related genes was also confirmed. To sum up, jumbo phage ZPAH34 was certified as a specific lytic phage with a safe profile that could be used as an antibacterial substitute in practical applications.

### 3.4. Morphology and biological characteristics of ZPAH34

**Figure 4:**
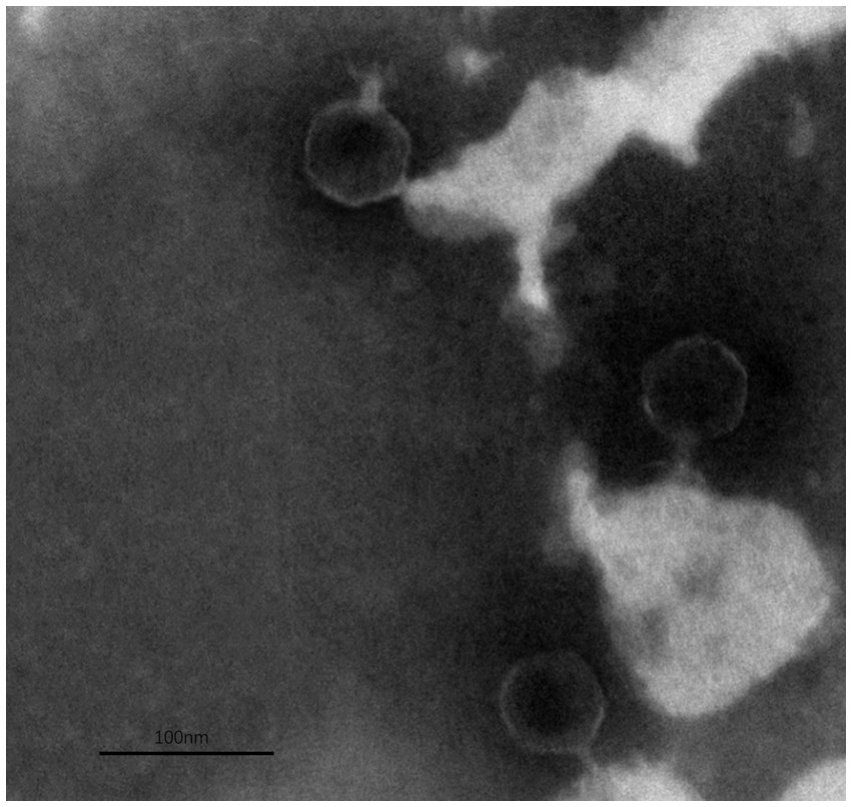
Transmission electron microscopy of ZPAH34.

**Figure 5:**
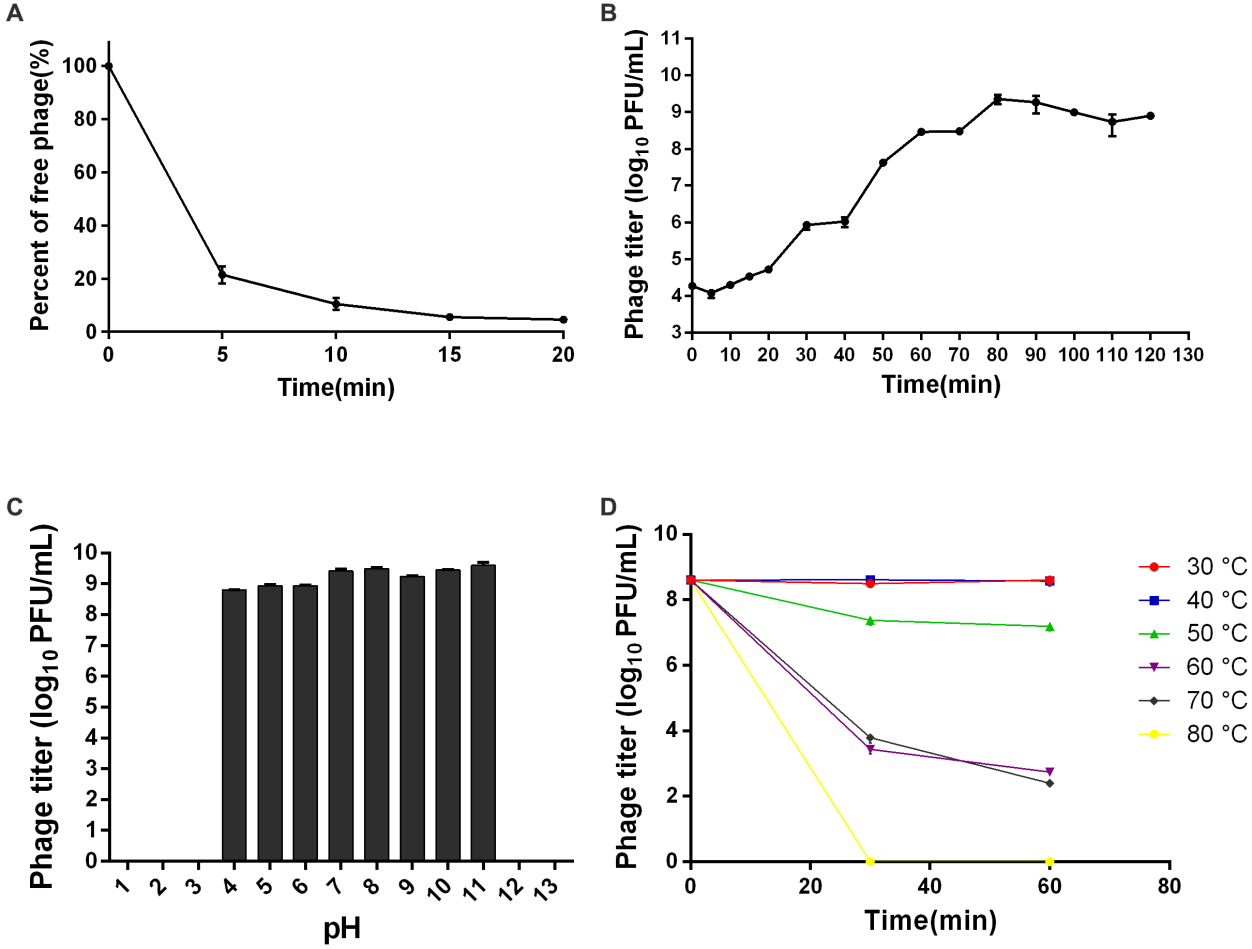
Biological properties of ZPAH34. **(A)** Adsorption assay of ZPAH34. **(B)** One-step growth curve of ZPAH34. **(C)** pH tolerance of ZPAH34. **(D)** Thermal tolerance of ZPAH34.

The TEM analysis revealed that phage ZPAH34 has an icosahedral head structure with a diameter of 53.4 ± 1.7 nm and a retractable tail with 22.5 ± 3 nm in size (Figure 4). Jumbo phages usually have big capsids to encapsulate their larger genomes compared to small-genome phages (Nazir et al., 2021). However, the phage ZPAH34 has the smallest capsid in comparison to other jumbo phages, according to the available morphology data of known jumbo phages (Supplementary Table S1). The optimal multiplicity of infection for phage ZPAH34 was 0.001, reaching a maximum potency of 4 × 10^8^ PFU/ml. The adsorption rate of phage ZPAH34 to the host bacteria reached 78.5% within 5 min, and 94.45% within 15 min (Figure 5A). The eclipse period, latent period and burst size are important indicator to determine the phage infection efficiency of bacteria. The one-step growth curve is shown in Figure 5B, the eclipse period of phage ZPAH34 was about 10 min, the latent period was 20 min, and burst size was 79 PFU/cell.

Furthermore, we evaluated the pH and thermal tolerances of ZPAH34. As shown in Figure 5C, phage ZPAH34 was incubated at pH 4–11 for 1 h and remained active with a titer of more than 10^8^ PFU/ml. While, bacteriophages could not survive in extremely acidic and alkaline environments at pH 2, 3, 12, and 13. Temperature tolerance assay shows in Figure 5D that phage ZPAH34 could maintain stable activity at 30–50°C, with almost constant titer. The phage count dropped to about 10^3^ PFU/ml after incubation at 60°C and 70°C, respectively.

### 3.5. Determining the taxonomic status of ZPAH34

To evaluate the taxonomic status of phage ZPAH34, all proteins were compared individually with other phages in the NCBI protein database using the BLASTP algorithm and the number of homologous proteins to ZPAH34 was counted (cover >60%, ident >35%). The established method for classifying virus into a separate genus is homologous protein >40% within a 75 bit as the BLASTP threshold by core gene site analysis (Lavigne et al., 2009). Protein homology analysis revealed that ZPAH34 was most closely related to phage CF8, PS1 and PS2 (Table 1), shared 75.8% homologous proteins with *Aeromonas* phage CF8 (MK774614), 57.32% homologous proteins with *Aeromonas* phage PS1 (MN032614) and 56.89% homologous proteins with *Aeromonas* phage PS2 (MN453779). The International Committee on Taxonomy of Viruses (ICTV) has not yet classify these phages. Meanwhile, CF8, PS1 and PS2 genomes were published without any further determination on their genera (Rai et al., 2020).

**Table 1:** Phages with homologous genes with ZPAH34 by BLASTP.

Phage name	Family /Subfamily	Genus	Genome length (kb)	Accession number	Homologous genes to ZPAH34	Homologous proteins rate	Reference
*Aeromonas phage CF8*	NA	NA	238.15	MK774614	176	75.8%	Rai et al., 2020
*Aeromonas phage PS1*	NA	NA	237.367	MN032614	133	57.32%	Rai et al., 2020
*Aeromonas phage PS2*	NA	NA	240.447	MN453779	132	56.89%	Rai et al., 2020
*Aeromonas phage LAh10*	NA	NA	260.31	MK838116	108	46.55%	Not published
*Klebsiella phage N1M2*	NA	NA	253.367	MN642089	103	44.39%	Lewis et al., 2020
*Erwinia phage AH04*	NA	NA	262.639	MZ501267	89	38.36%	Krukonis et al., 2021
*Aeromonas phage D3*	NA	NA	262.372	MN102098	85	36.63%	Not published
*Edwardsiella virus pEtSU*	NA	Petsuvirus	276.734	NC_048182	84	36.2%	Kim et al., 2019
*Pseudomonas phage OBP*	NA	Petsuvirus	284.757	JN627160	78	33.62%	Semenyuk et al., 2016
*Aeromonas phage D6*	NA	NA	259.831	MN131137	57	24.56%	Not published
*Klebsiella phage Miami*	NA	NA	253.383	MT701590	51	21.98%	Mora et al., 2021
*Erwinia phage vB EamM_RisingSun*	NA	Risingsunvirus	235.018	MF459646	44	18.97%	Arens et al., 2018
*Aeromonas phage D9*	NA	NA	260.857	MN159079	39	16.81%	Not published
*Vibrio phage 2 TSL-2019*	Gorgonvirinae	Aphroditevirus	242.446	MK368614	36	11.52%	Not published
*Vibrio phage pTD1*	Gorgonvirinae	Tidunavirus	239.276	AP017972	35	15.09%	Not published
*Vibrio phage USC-1*	Gorgonvirinae	Aphroditevirus	238.099	MK905543	29	12.5%	Not published
*Pseudomonas phage EL*	NA	Elvirus	211.215	AJ697969	28	12.07%	Hertveldt et al., 2005
*Photobacterium phage PDCC-1*	Gorgonvirinae	Aphroditevirus	237509	MN562221	28	12.07%	Not published
*Vibrio phage VP4B*	Gorgonvirinae	Tidunavirus	236.053	KC131130	27	11.63%	Not published
*Erwinia phage vB_EamM_Joad*	NA	Risingsunvirus	235.374	MF459647	22	9.48%	Arens et al., 2018
*Vibrio phage Aphrodite1*	Gorgonvirinae	Aphroditevirus	237.722	MG720308	21	9.05%	Not published
*Aeromonas phage AP1*	Emmerichvirin	Ceceduovirus	254.49	MT713136	5	2.16%	Hassan et al., 2018
*Erwinia phage Ea35-70*	NA	Agricanvirus	271.084	KF806589	4	1.72%	Not published
*Erwinia phage pEa_SNUABM_37*	NA	NA	294.404	MW845760	3	1.29%	Not published
*Proteus phage 10*	NA	NA	223.209	MT661596	2	0.86%	Not published
*Salmonella phage vB_SalM_SA002*	NA	NA	288.012	MN445183	2	0.86%	Not published
*Klebsiella phage vB_KvM-Eowyn*	NA	NA	265.389	LR881104	2	0.86%	Not published
*Xanthomonas phage Xoo-sp14*	NA	NA	232.104	MT939492	2	0.86%	Not published
*Xanthomonas phage vB LucasX*	NA	NA	305.651	MW825358	2	0.86%	Not published

NA, not available.

**Figure 6:**
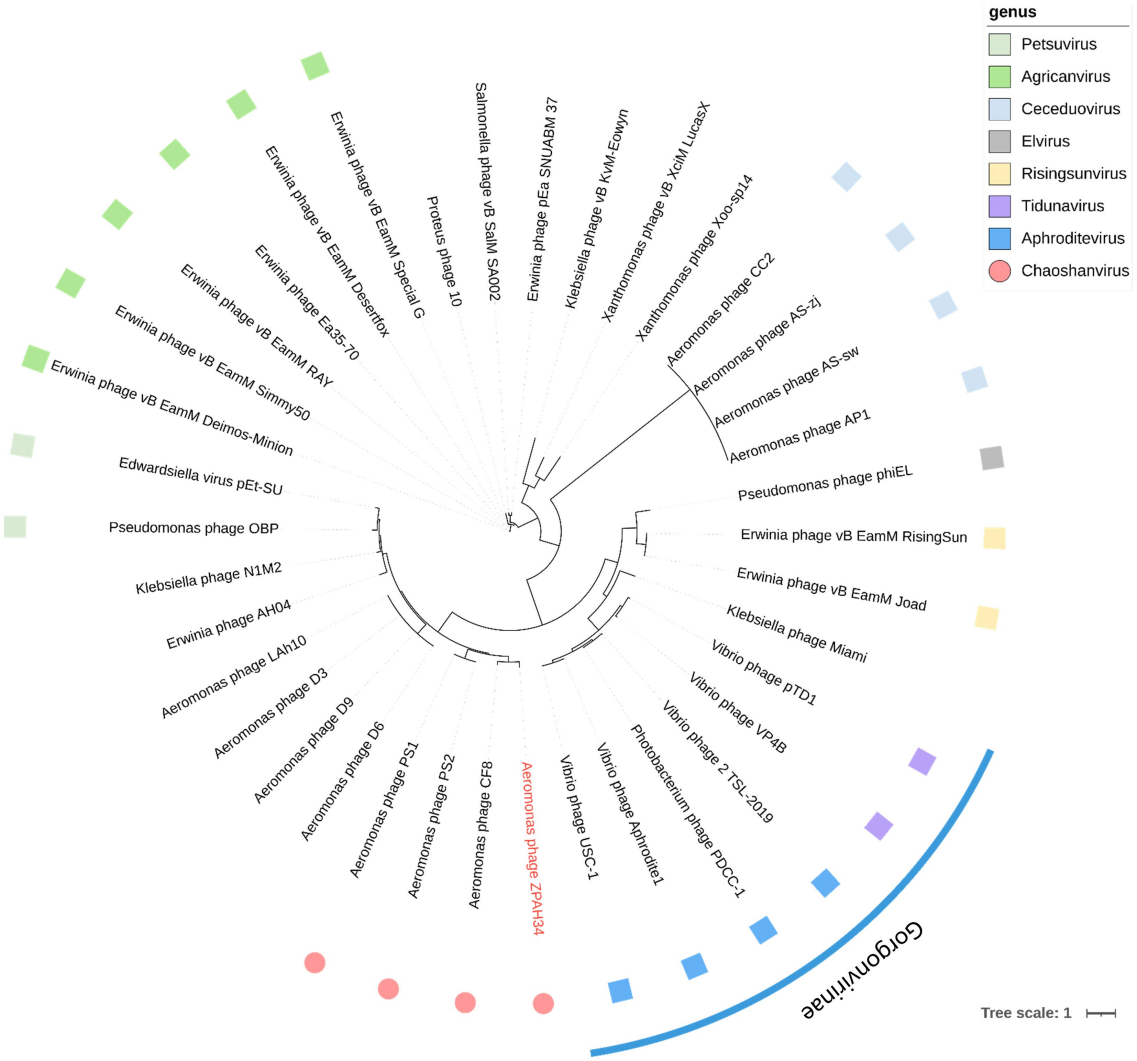
Phylogenetic tree based on their major capsid proteins was constructed using IQ-tree. The blue arc is used to indicate phages that are classified as the same subfamily. The square of the same color represents phages classified as the same genus. The roundness indicates the phages of the new proposed genus *Chaoshanvirus*.

To further verify whether these four phages are distinct from other genera in terms of taxonomic status, phylogenetic analysis based on the major capsid protein, which is conserved in genetic evolution (Hatfull and Hendrix, 2011), was conducted. Based on the classification of viruses in the ICTV, all phages which have homologous proteins with ZPAH34, and other members of their genera were collected. Phylogenetic analysis indicated that ZPAH34, CF8, PS1 and PS2 belonged to a clade that separated from the other genera (Figure 6), which was consistent with the results of the homologous protein analysis. The results suggested that these four phages may represent a new genus, which was named *Chaoshanvirus*.

### 3.6. Evaluation of bactericidal capacity of phage ZPAH34 in food

**Figure 7:**
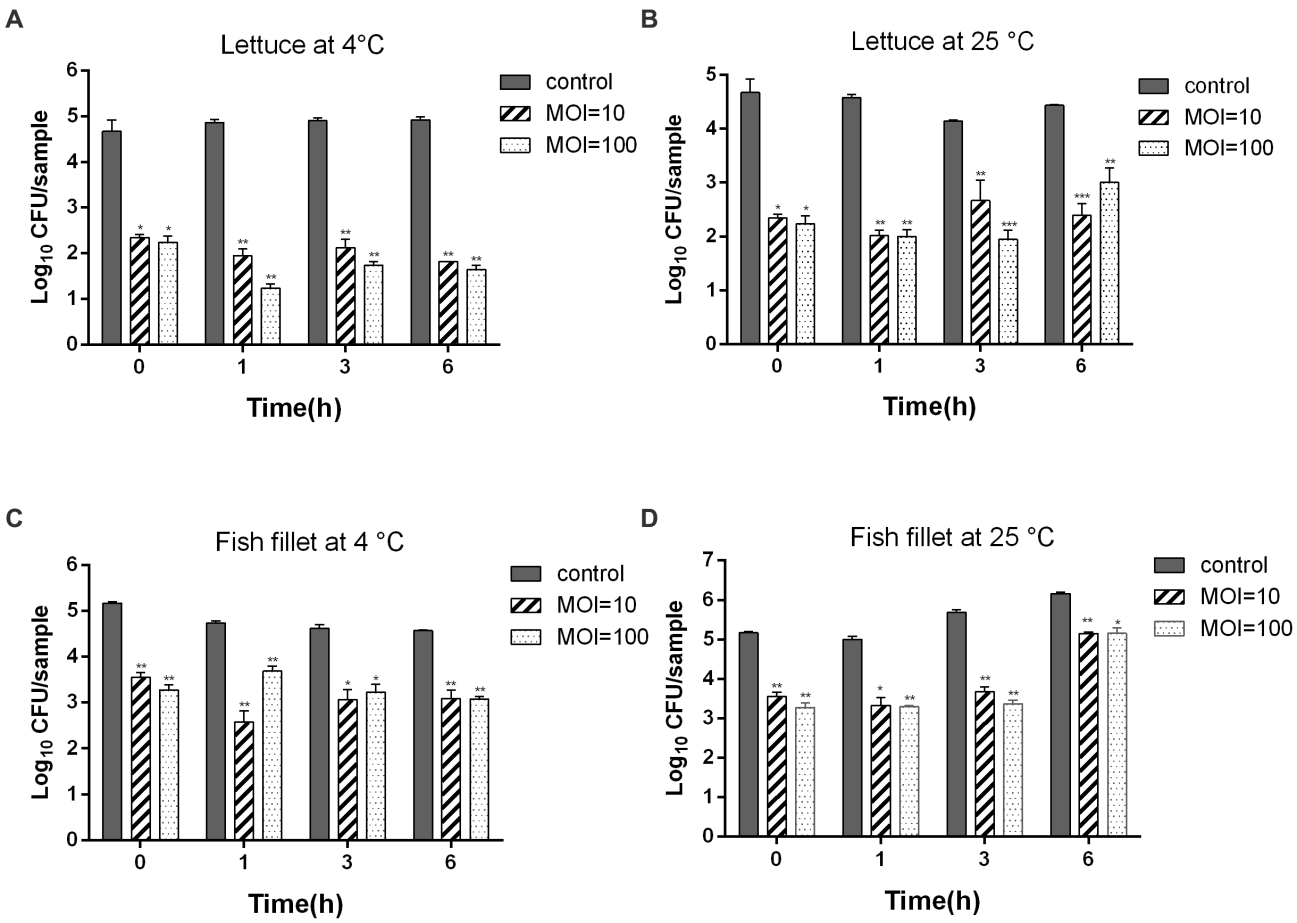
Inhibition of ZPAH34 against *A. hydrophila* ZYAH75 in lettuce incubated at 4°C **(A)** and 25°C **(B)** and fish fillet incubated at 4°C **(C)** and 25°C **(D)**. Error bars represent the mean ± SD. *p < 0.05; **p < 0.01.

To assess whether ZPAH34 is a candidate for biocontrol, its bactericidal ability was measured on lettuce and fish fillets at 4°C and 25°C. As shown in Figure 7A, phage ZPAH34 considerably inhibited the growth of *A. hydrophila* on lettuce at 4°C, the viable count reduced by 3.14 log CFU/sample with an MOI of 10 and 3.28 log CFU/sample with an MOI of 100 after 6 h. When incubated under room temperature, after incubation with an MOI of 10 and 100 for 6 h, the bacteria decreased significantly by 2.05 log CFU/sample and 1.46 log CFU/sample, respectively (Figure 7B).

Similar antimicrobial effects were observed in fish fillets samples, 1.48 and 1.49 log CFU/sample reductions (*p* < 0.05) were discovered at an MOI of 10 and 100 after 4°C storage for 6 h, respectively (Figure 7C). When fish fillets storage at 25°C, phage ZPAH34 treatment greatly declined the *A. hydrophila* cell of 1.84, 2, and 1.01 log CFU/sample at an MOI = 10 and of 1.71, 2.31 and 1 log CFU/sample with MOI = 100 at 1, 3, and 6 h, respectively (*p* < 0.05) (Figure 7D). These results indicated that phage ZPAH34 can effectively control the contamination of foodborne pathogens.

## 4. Discussion

*Aeromonas hydrophila*, widespread in water, water habitats, domestic animals, and foods (fish, shellfish, poultry, raw meat, vegetables, and dairy products), has the potential to be a foodborne pathogen (Igbinosa et al., 2012; Park et al., 2021). *A. hydrophila* residues on food surfaces or in processing and storage can increase the risk of food spoilage (Stratev and Odeyemi, 2016), pose a threat to human health. Moreover, several studies have shown that the formation of biofilms and the emergence of antibiotics resistance in *A. hydrophila* make it more challenging to control *A. hydrophila* contamination in food (Tipmongkolsilp et al., 2012). Phage, as an alternative antimicrobial agent, is a candidate for effective control of foodborne pathogenic bacteria in food industry.

Considering the bactericidal efficiency of phages is an essential feature as a biocontrol agent. First, the lytic activities of phage isolates were tested. Phage ZPAH34 could completely inhibit the growth of *A. hydrophila* within 12 h, showing significant lytic ability compared to phages ZPAH12, ZPAH21, ZPAH29, ZPAH71, ZPAH85, ZPAH103, ZPAH106, ZPAH109, and ZPAH118. Consequently, we selected ZPAH34 to test its bactericidal effect on host strain biofilms. Bacterial biofilms are structured communities formed in extracellular polymeric substances attached to the surface of organisms that protect bacteria from environmental stress and resist erosion by the host immune system, thereby increasing the risk of bacterial resistance and cross-resistance (Abdi-Ali et al., 2006; Barger et al., 2021). Biofilms adhering to the surfaces of various tissues and materials become a major factor of chronic infection and drug resistance. (Du et al., 2016). Biofilm-associated *A. hydrophila* may have an advantage over planktonic ones in attachment and invasion (Barger et al., 2021). Phage have been proved to effectively reduce the biofilms, phage ZPAH7 could produce polysaccharide depolymerases to degrade MDR *A. hydrophila* biofilms on two abiotic surfaces (Islam et al., 2021). The tail spike protein of phage vB_PmiS_PM-CJR has a pectate lyase domain exerting lytic activity to reduce *Proteus mirabilis* biofilms (Rice et al., 2021). In this study, phage ZPAH34 has effects in both inhibiting biofilm formation and degrading mature biofilms established on the surface of different materials. The application of a phage inhibited biofilms in 96-well microplates (75.76–76.35%) and on glass surfaces (ranging from 1.35 to 1.77 log CFU/ml). Biofilms adhering to the 96-well microplate and glass were effectively degraded by phage ZPAH34 at a titer of 10^8^ PFU/ml or 10^9^ PFU/ml. Our results suggest that phage ZPAH34 has potential to control biofilms formed by *A. hydrophila* on food contact surfaces.

It was found that phage ZPAH34 showed the highest bactericidal capacity at an MOI of 0.001. Phage ZPAH34 has a burst size of 79 ± 5 PFU/host cell and a short latent period, which allows it to multiply quickly and release phage particles to obtain a greater bactericidal effect. Compared to previous studies, the adsorption rate of ZPAH34 was faster than other *Aeromonas* phage (Anand et al., 2016; Cheng et al., 2021). Phages are subject to various physicochemical environmental influences as natural antimicrobial agents, therefore the stability of phages under various environmental stress is an indicator to assess whether phages can be used as biocontrol agents in food (Abedon and Culler, 2007; Tomat et al., 2014). Phage ZPAH34 showed broad tolerance to diverse physicochemical environments, maintaining a relatively stable activity in the temperature range of 30–50°C and pH range of 4–11. According to previous reports of *Aeromonas* phages, AhyVDH1 was stable only in the pH range of 5–10, and the survival rate decreased to 66.7 and 24.3% when treated at 40 and 50°C, respectively (Cheng et al., 2021). The activity of AHP-1 decreased to 25% at 50°C (Chandrarathna et al., 2020). Therefore, the tolerance of heat and pH of phage ZPAH34 makes it more applicable in food matrix.

Jumbo phage ZPAH34 consists of an icosahedral head and contractile tail identified by transmission electron microscopy. Previous studies have suggested that jumbo phages with large genome size (>200 kb) also have fairly large structural dimensions (Supplementary Table S1), but the viral dimension identified in our study is the smallest one in the jumbo phages reported to date, and the smaller capsid allows for a more tightly wrapped genome (Thomas et al., 2016). Genomic analysis revealed that the genome length of ZPAH34 is 234 kb. It is known that jumbo phages possess many auxiliary genes compared to small phages (Malone et al., 2020). A total of 80/234 ORFs of ZPAH34 encode different functional proteins, and the remaining 154 ORFs lacking sequence similarity to other phage sequences in the GenBank were classified as hypothetical protein-encoding genes. We identified two endolysins (ORF38, ORF128) involved in bacterial lysis and two tRNAs implemented an effective transcriptional strategy. One endolysin belonging to transglycosylase degrades bacterial peptidoglycan via cleavage of the β-1,4 glycosidic bond to promote infection (Briers, 2019). In jumbo phages, there is more than one paralogous gene for DNA polymerase and RNA polymerase (RNAP) which are most multi-subunit RNAPs (Thanki et al., 2019). We identified genes encoding multi-subunit RNAPs in the jumbo phage ZPAH34 that are able to mediate early gene transcription during infection and are not dependent on the host RNAPs (Iyer et al., 2021). The ‘phage nucleus’ is formed micrometer-scale compartments in host which functions to segregated viral DNA and bacterial proteins, and protect jumbo phage from broadly host immunity during the early stages of assembly (Chaikeeratisak et al., 2017; Laughlin et al., 2022). We predicted a nuclear shell protein, chimallin, encoded by gene 210. Recent studies have revealed that the chimallin protein of jumbo phage 201phi2-1 self-assembled into a protective compartment, that precludes host CRISPR-Cas systems and restriction enzymes yet allowing involved in DNA replication and transcription expressed proteins transport (Laughlin et al., 2022). Similarly assembled nuclear shell morphologies were observed in the jumbo phages Goslar, PhiPA3, and PhiKZ15 (Birkholz et al., 2022). Furthermore, the gene encoding the chimallin homolog was found in most of the jumbo phage, suggesting a similar protective mechanism may exist in jumbo phages early in infection. Genome analysis of the lytic phage ZPAH34 showed that no genes involved in lysogenic conversion were detected. Meanwhile, there was no gene associated with antibiotic resistance, and toxin and pathogenicity factors. Our studies demonstrate the safety of ZPAH34 in food applications (Endersen and Coffey, 2020).

A cut-off value of 40% homologous proteins within 75 positions by core locus analysis was used to identify a new phage genera (Lavigne et al., 2009). Phage CF8, PS1 and PS2 isolated from water samples in India have high homology with ZPAH34. Their geographical dispersion, but the high similarity to each other, can be explained by their complex coevolution with the host bacterial strains (Markwitz et al., 2022). Based on phylogenetic analysis of the protein sequences of the major capsid protein, these four phages clustered together to form a separate clade, distinguishing them from other clades. Therefore, we classified them into a new genus named *Chaoshanvirus*. The increasing number of new jumbo phage sequences allows genomic analysis to precisely define new genera and subfamilies of phages and to better refined to viral taxonomy (Lavigne et al., 2009; Dion et al., 2020).

Previous studies have revealed that phages as biocontrol agents are effective in reducing bacterial contamination, such as phage fmb-p1 (9.9 × 10^9^ PFU/cm^2^) reduced the viable count of *Salmonella Typhimurium* in ready-to-eat duck meat (Wang et al., 2017). Phage cocktail significantly reduced *Salmonella Enteritidis* and *Salmonella Typhimurium* in raw chicken breast (Duc et al., 2018). In this study, we evaluated the bactericidal ability of phage ZPAH34 on lettuce. The application of ZPAH34 to bacteria-contaminated lettuce leaves at 4 and 25°C. After incubation for 6 h, *A. hydrophila* on the samples decreased by 1.46 log to 3.28 log CFU/sample. Researchers applied a phage to raw fish flesh slices to reduce *Vibrio parahaemolyticus* 3.9 log, but no studies have reported the control effect of phage against *A. hydrophila* on fish fillets (You et al., 2021). Our results showed that phage ZPAH34 strongly inhibited the bacterial growth at different temperatures, reducing the number of bacteria by 1 to 2.31 log CFU/sample. In general, phage ZPAH34 could effectively inhibit pathogenic bacteria *A. hydrophila* ZYAH75 in food matrices with low MOI and prolong food freshness with high potential for food application (O’Sullivan et al., 2019).

In this study, a lytic jumbo phage ZPAH34 was isolated and characterized. The phage was able to inhibit the growth of MDR *A. hydrophila* and prevented bacterial biofilm contamination. It exhibited a wide range of pH and temperature tolerance, with a fast adsorption rate and a large burst size. Gene sequence analysis indicated that ZPAH34 is a new jumbo phage possessing minimum virions. Further phylogenetic analysis showed that ZPAH34 was clustered in a clade with three other phages, belonging to a new genus named *Chaoshanvirus*. No virulence genes or drug resistance genes were found in the ZPAH34 genome. Accordingly, this study presented a renewed approach using phage therapy to combat MDR bacteria in food matrix, which could be implemented with other common contamination abatement/prevention methods to eliminate the bacterial contamination thoroughly in the food sector.

## Data availability statement

The datasets presented in this study can be found in online repositories. The names of the repository/repositories and accession number(s) can be found in the article/Supplementary material.

## Author contributions

YZ conceived and designed the experiments. YH, ZW, LR, and YC performed the experiments. YH contributed to data curation. YH and YZ wrote the original draft. Y-AZ and YZ wrote, review and edited the manuscript. All authors have read and agreed to the published version of the manuscript.

## Funding

The study was supported by the National Natural Science Foundation of China (32073022), the China Agriculture Research System of MOF and MARA (CARS-46), the Laboratory of Lingnan Modern Agriculture Project (NT2021008), and HZAU-AGIS Cooperation Fund (SZYJY2022027).

## Conflict of interest

The authors declare that the research was conducted in the absence of any commercial or financial relationships that could be construed as a potential conflict of interest.

## Publisher’s note

All claims expressed in this article are solely those of the authors and do not necessarily represent those of their affiliated organizations, or those of the publisher, the editors and the reviewers. Any product that may be evaluated in this article, or claim that may be made by its manufacturer, is not guaranteed or endorsed by the publisher.

## Supplementary material

The Supplementary material for this article can be found online at: https://www.frontiersin.org/articles/10.3389/fmicb.2023.1178876/full#supplementary-material

## Footnotes

^1^ https://www.ncbi.nlm.nih.gov

^2^ https://card.mcmaster.ca/

^3^ http://www.mgc.ac.cn/VFs/main.htm
