# Supplementary material for: Characterization and application of a lytic jumbo phage ZPAH34 against multidrug-resistant Aeromonas hydrophila
Source: Front Microbiol. 2023 Jun 21;14:1178876. doi: 10.3389/fmicb.2023.1178876 (PMC10321303; doi:10.3389/fmicb.2023.1178876)
Supplement: Supplementary file 1 [file Table_1.docx]

Supplementary Material

Characterization and application of a lytic jumbo phage ZPAH34 against multidrug-resistant *Aeromonas hydrophila*

Yuting Hou ^1^, Yong-An Zhang^*^, Yang Zhou^*^

*** Correspondence:**Y.-A. Zhang: yonganzhang@mail.hzau.edu.cn

Y. Zhou: zhouyang@mail.hzau.edu.cn

# Supplementary Table

**Table S1.** General features of jumbo phages

| Phage | | | Host | Virion size (nm) | | | | Genome length (nt) | No. of CDSs | No. of tRNA | | References |
| --- | --- | --- | --- | --- | --- | --- | --- | --- | --- | --- | --- | --- |
|  | | |  | Head | | Tail length | |  |  |  | |  |
| G | | | *Bacillus megaterium* | 160 | | 453 | | 497,513 | 675 | 20 | Donelli et al., 1975 | |
| Atu_ph07 | | | *Agrobacterium tumefaciens* | 145×152 | | 136 | | 490,380 | 714 | 33 | Attai et al 2018 | |
| XacN1 | | | *Xanthomonas citri* | NA | | NA | | 384,670 | 592 | 56 | Yoshikawa et al 2018 | |
| vB_Pca_CBB | | | *Enterobacteria* | 126.9 | | 123 | | 378,378 | 554 | 33 | Buttimer et al 2017 | |
| ΦMV-4 | | | *Morganella morganii* | 307 | | 122 | | 360,000 | NA | NA | Yamaki et al 2019 | |
| vB_CsaM_GAP32 | | | *Cronobacter sakazakii* | 115 | | 118 | | 358,663 | 545 | 26 | Abbasifar et al 2014 | |
| pEa_SNUABM_12 | | | *Erwinia amylovora* | 130±5.9 | | 126.7±2.6 | | 358,115 | 546 | 32 | Kim et al 2020 | |
| pEa_SNUABM_50 | | | *Erwinia amylovora* | 130±5.9 | | 126.7±2.6 | | 356,948 | 540 | 35 | Kim et al 2020 | |
| pEa_SNUABM_47 | | | *Erwinia amylovora* | 130±5.9 | | 126.7±2.6 | | 355,376 | 540 | 34 | Kim et al 2020 | |
| phAPEC6 | | | *Escherichia coli* | 136 | | NA | | 350,175 | 551 | NA | Wagemans et al 2020 | |
| Munch | | | *Salmonella* | 119 × 99 | | 125 × 23 | | 350,103 | 532 | 22 | Yicheng Xie et al 2021 | |
| 121Q | | | *Escherichia coli* | 116 | | 115 | | 348,532 | 611 | 7 | Ackermann et al 1983 | |
| PBECO 4 | | | *Escherichia coli* | 132 | | 125 | | 348,113 | 551 | 6 | Kim et al 2013 | |
| Phage | | | Host | Virion size (nm) | | | Genome length (nt) | | No. of CDSs | No. of tRNA | | References |
|  | | |  | Head | Tail length | |  | |  |  | |  |
| GBH019 | | | *Klebsiella pneumoniae* | 132.7 | | 163.4 | 347,546 | | 534 | 6 | Blundell-Hunter et al 2021 | |
| Muenster | | | *Klebsiella pneumoniae* | NA | | NA | 346,937 | | 561 | 6 | Martin et al 2021 | |
| K64-1 | | | *Klebsiella pneumoniae* | NA | | NA | 346,602 | | 64 | NA | Pan et al 2015 | |
| vB_KleM-RaK2 | | | *Klebsiella sp.* | 123 | | 128 | 345,809 | | 534 | 5 | Šimoliūnas et al 2013 | |
| RsoM2USA | | | *Ralstonia solanacearum* | 142 | | 12 | 343,806 | | 486 | 44 | Ahmad et al 2021 | |
| 201phi2-1 | | | *Pseudomonas chlororaphis* | 129 | | 200 | 316,674 | | 461 | 1 | Thomas et al 2008 | |
| PhiPA3 | | | *Pseudomonas aeruginosa* | 100 | | 185 | 309,208 | | 375 | 5 | Monson et al 2011 | |
| Phabio | | | *Pseudomonas fluorescens* | 134 | | 174 | 309,157 | | 469 | 3 | Joanna et al 2017 | |
| Xoo-sp13 | | | *Xanthomonas oryzae pvoryza* | 60 | | 121 | 309,023 | | 401 | 28 | Nazir et al 2020 | |
| ΦKp34 | | | *Klebsiella pneumoniae:* | 110 | | 177 | 307,210 | | 372 | 9 | Bonilla et al 2021 | |
| vb_XciM_LucasX | | | *Xanthomonas citri* | NA | | 325 | 305,651 | | 394 | 5 | Marquioni et al 2022 | |
| Psa21 | | | *Pseudomonas syringae* | NA | | NA | 305,260 | | 420 | 8 | Frampton et al 2019 | |
| Bonaishi | | | *Vibrio coralliilyticus* | 120 | | 190 | 303,340 | | 301 | 0 | Jacquemot et al 2018 | |
| MIJ3 | | | *Pseudomonas aeruginosa* | 130 | | 140 | 288,170 | | 417 | 12 | Imam et al 2019 | |
| Phage | | | Host | Virion size (nm) | | | Genome length (nt) | | No. of CDSs | No. of tRNA | | References |
|  | | |  | Head | Tail length | |  | |  |  | |  |
| PA5oct | | | *Pseudomonas aeruginosa* | 131 | | 136 | 286,783 | | 465 | 12 | Cédric Lood et al 2020 | |
| vB_VhaM_pir03 | | | *Vibrio harveyi* | 132×123 | | 247 | 286,284 | | 334 | 0 | Misol et al 2020 | |
| OBP | | | *Pseudomonas fluorescens* | 119 | | 191 | 284,757 | | 309 | 4 | Cornelissen et al 2012 | |
| ɸU5 | | | *Pseudomonas aeruginosa* | 121 | | 210 | 282,684 | | 373 | 6 | Rai et al 2022 | |
| Lu11 | | | *Pseudomonas putida* | 124 | | 200 | 280,538 | | 391 | 0 | Adriaenssens et al 2012 | |
| phiKZ | | | *Pseudomonas aeruginosa* | 145 | | 160 | 280,334 | | 306 | 6 | Mesyanzhinov et al | |
| Noxifer | | | *Pseudomonas fluorescens* | 130 | | 191 | 278,136 | | 334 | 4 | Joanna et al 2017 | |
| CcrColossus | | | *Caulobacter crescentus* | 292×95 | | 65 | 279,967 | | 448 | 28 | Meczker et al 2014 | |
| RP12 | | | *Ralstonia solanacearum* | 120 | | 180 | 279,845 | | 289 | 1 | Matsui et al 2017 | |
| KTN4 | | | *Pseudomonas aeruginosa* | 130 | | 168 | 279,593 | | 368 | 6 | Danis-Wlodarczyk et al 2016 | |
| RP31 | | | *Ralstonia solanacearum* | 120 | | 180 | 276,958 | | 287 | 1 | Matsui et al 2017 | |
| Machias | | | *Staphylococcus aureus* | NA | | NA | 274,478 | | 263 | 13 | Korn et al 2021 | |
| PhiMa05 | | | *Microcystis* | 100 | | 120 | 273,876 | | 254 | 6 | Naknaen et al 2021 | |
| vB_EamM_Deimos Minion | | | *Erwinia amylovora* | NA | | NA | 273,501 | | 326 | NA | Esplin et al 2017 | |
| Phage | | | Host | Virion size (nm) | | | Genome length (nt) | | No. of CDSs | No. of tRNA | | References |
|  | | |  | Head | Tail length | |  | |  |  | |  |
| vB_EamM_Special G | | | *Erwinia amylovora* | NA | | NA | 273,224 | | 324 | 0 | Esplin et al 2017 | |
| vB_EamM_RAY | | | *Erwinia amylovora* | NA | | NA | 271,182 | | 319 | 1 | Esplin et al 2017 | |
| vB_EamM_Simmy 50 | | | *Erwinia amylovora* | NA | | NA | 271,088 | | 321 | 1 | Esplin et al 2017 | |
| Ea35-70 | | | *Erwinia amylovora* | NA | | NA | 271,084 | | 318 | 1 | Yagubi et al 2014 | |
| PALS2 | | | *Staphylococcus aureus* | 101 | | 201 | 268,746 | | 279 | 1 | Lee et al 2021 | |
| PA7 | | | *Pseudomonas aeruginosa* | NA | | NA | 266,743 | | 341 | NA | Kwan et al 2006 | |
| MarsHill | | | *Staphylococcus aureus* | 115 | 233 | | 266,637 | | 262 | 9 | Korn et al 2021 | |
| Madawaska | | | *Staphylococcus aureus* | NA | | NA | 265,446 | | 264 | 10 | Korn et al 2021 | |
| phiR1-37 | | | *Yersinia enterocolitica* | 138 | 383 | | 262,391 | | 367 | 5 | Kiljunen et al 2005 | |
| AD1 | | | *Dickeya solani* | NA | NA | | 261,658 | | 332 | 0 | Day et al 2018 | |
| vB_EamM_Y3 | | | *Erwinia amylovora* | NA | NA | | 261,365 | | 338 | 0 | Buttimer et al 2018 | |
| vB_StaM_SA1 | | | *Staphylococcus aureus* | 100 | 220 | | 260,727 | | 258 | 1 | Bingyan Zhang et al 2022 | |
| vB_EamM_Yoloswag | | | *Erwinia amylovora* | NA | NA | | 259,700 | | 334 | NA | Esplin et al 2017 | |
| PaBG | | | *Pseudomonas aeruginosa* | 136 | 220 | | 258,139 | | 308 | NA | Sykilinda et al 2014 | |
| Phage | | | Host | Virion size (nm) | | | Genome length (nt) | | No. of CDSs | No. of tRNA | | References |
|  | | |  | Head | Tail length | |  | |  |  | |  |
| vB_BpuM_BpSp | | | *Bacillus pumilus* | 137 | 192 | | 255,569 | | 318 | 0 | Yuan et al 2016a, | |
| JA13 | | | *Dickeya solani* | NA | NA | | 254,061 | | 326 | 0 | Day et al 2018 | |
| JA29 | | | *Dickeya solani* | NA | NA | | 253,323 | | 324 | 0 | Day et al 2018 | |
| N1M2 | | | *Klebsiella aerogenes* | 113 | 158 | | 253,367 | | 257 | 24 | Rhea Lewis et al 2020 | |
| JA11 | | | *Dickeya solani* | NA | NA | | 255,356 | | 325 | 0 | Day et al 2018 | |
| JA33 | | | *Dickeya solani* | NA | NA | | 255,356 | | 325 | 0 | Day et al 2018 | |
| vB-AhyM-AP1 | | | *Aeromonas hydrophila* | NA | NA | | 254,490 | | 455 | 22 | Pallavi et al 2021 | |
| P-SSM2 | | | *Prochlorococcus* | 115 | 123 | | 252,401 | | 334 | 1 | Sullivan et al 2005 | |
| AR9 | | | *Bacillus subtilis* | NA | NA | | 251,042 | | 291 | 1 | Lavysh et al 2016 | |
| ValKK3 | | | *Vibrio alginolyticus* | NA | NA | | 248,088 | | 390 | NA | Lal et al 2016 | |
| nt-1 | | | *Vibrio natriegens* | NA | NA | | 247,511 | | 405 | 28 | Comeau et al 2014 | |
| Va3 | | | *Vibrio alginolyticus* | 107 | 95 | | 247,567 | | 34 | 383 | Chengcheng Li et al 2022 | |
| VH7D | | | *Vibrio harveyi* | NA | NA | | 246,964 | | 327 | NA | Luo et al 2015 | |
| phi-pp2 | | | *Vibrio parahaemolyticus* | 90×50 | 110 | | 246,421 | | 383 | 30 | Lin and Lin 2012 | |
| Phage | | | Host | Virion size (nm) | | | Genome length (nt) | | No. of CDSs | No. of tRNA | | References |
|  | | |  | Head | Tail length | |  | |  |  | |  |
| vB_EamM_Kwan | | | *Erwinia amylovora* | NA | NA | | 246,390 | | 285 | 8 | Esplin et al 2017 | |
| vB_EamM_Asesino | | | *Erwinia amylovora* | NA | NA | | 246,291 | | 277 | NA | NA | |
| vB_EamM_ChrisDB | | | *Erwinia amylovora* | NA | NA | | 244,840 | | 277 | 11 | Esplin et al 2017 | |
| KVP40 | | | *Vibrio parahaemolyticus* | 140×70 | NA | | 244,834 | | 381 | 30 | Miller et al 2003 | |
| phiEaH2 | | | *Erwinia amylovora* | NA | NA | | 243,050 | | 262 | NA | Dömötör et al 2012 | |
| vB_EamM_Stratton | | | *Erwinia amylovora* | NA | NA | | 243,953 | | 276 | 12 | Esplin et al 2017 | |
| SPFM1 | | | *Salmonella* *Typhimurium* | 100 | 160 | | 242,624 | | 307 | 1 | Thanki et al 2019 | |
| vB_EamM_Machina | | | *Erwinia amylovora* | NA | NA | | 241,654 | | 272 | 9 | Esplin et al 2017 | |
| SPFM13 | | | *Salmonella* *Typhimurium* | 135 | 140 | | 241, 405 | | 261 | 1 | Thanki et al 2019 | |
| vB_EamM_Caitlin | | | *Erwinia amylovora* | NA | NA | | 241,147 | | 271 | 7 | Esplin et al 2017 | |
| vB_EamM_Parshik | | | *Erwinia amylovora* | NA | NA | | 241,050 | | 271 | 10 | Esplin et al 2017 | |
| vB_EamM_Huxley | | | *Erwinia amylovora* | NA | NA | | 240,761 | | 271 | 9 | Esplin et al 2017 | |
| SPN3US | | | *Salmonella enterica* | NA | NA | | 240,413 | | 264 | 2 | Lee et al 2011 | |
| vB_EcoM_EC001 | | | *Escherichia coli* | 120 | 190 | | 240,200 | | 270 | 1 | Cucic et al 2022 | |
| Phage | | Host | | Virion size (nm) | | | Genome length (nt) | | No. of CDSs | No. of tRNA | | References |
|  |  | | | Head | Tail length | |  | |  |  | |  |
| SPFM3 | | | *Salmonella* *Typhimurium* | 105 | 200 | | 240,198 | | 257 | 1 | Thanki et al 2019 | |
| SPFM8 | | | *Salmonella* *Typhimurium* | 105 | 200 | | 240,197 | | 305 | 1 | Thanki et al 2019 | |
| SPFM12 | | | *Salmonella* *Typhimurium* | 100 | 160 | | 240,197 | | 305 | 1 | Thanki et al 2019 | |
| SPFM6 | | | *Salmonella* *Typhimurium* | 100 | 160 | | 240, 197 | | 298 | 1 | Thanki et al 2019 | |
| SPFM9 | | | *Salmonella* *Typhimurium* | 105 | 200 | | 240,197 | | 298 | 1 | Thanki et al 2019 | |
| SPFM7 | | | *Salmonella* *Typhimurium* | 100 | 160 | | 240,197 | | 290 | 1 | Thanki et al 2019 | |
| SPFM14 | | | *Salmonella* *Typhimurium* | 100 | 160 | | 240,197 | | 289 | 1 | Thanki et al 2019 | |
| SPFM10 | | | *Salmonella* *Typhimurium* | 100 | 160 | | 240,197 | | 285 | 1 | Thanki et al 2019 | |
| SPFM19 | | | *Salmonella* *Typhimurium* | 105 | 200 | | 240, 197 | | 258 | 1 | Thanki et al 2019 | |
| SPFM4 | | | *Salmonella* *Typhimurium* | 105 | 200 | | 240, 197 | | 257 | 1 | Thanki et al 2019 | |
| SPFM11 | | | *Salmonella* *Typhimurium* | 105 | 200 | | 240, 197 | | 257 | 1 | Thanki et al 2019 | |
| SPFM21 | | | *Salmonella* *Typhimurium* | 105 | 200 | | 240,196 | | 259 | 1 | Thanki et al 2019 | |
| SPFM22 | | | *Salmonella* *Typhimurium* | 100 | 160 | | 240,196 | | 259 | 1 | Thanki et al 2019 | |
| SPFM5 | | | *Salmonella* *Typhimurium* | 105 | 200 | | 240,194 | | 287 | 1 | Thanki et al 2019 | |
| Phage | | Host | | Virion size (nm) | | | Genome length (nt) | | No. of CDSs | No. of tRNA | | References |
|  |  | | | Head | Tail length | |  | |  |  | |  |
| SPFM2 | | *Salmonella* *Typhimurium* | | 100 | 160 | | 240,111 | | 260 | 1 | Thanki et al 2019 | |
| SPFM15 | | *Salmonella* *Typhimurium* | | 100 | 160 | | 239,951 | | 289 | 1 | Thanki et al 2019 | |
| SPFM17 | | *Salmonella* *Typhimurium* | | 100 | 160 | | 239,842 | | 258 | 1 | Thanki et al 2019 | |
| pSal-SNUABM-04 | | *Salmonella sp* | | 80 | 116 | | 239,626 | | 280 | 10 | Kwon et al 2020 | |
| SPFM20 | | *Salmonella* *Typhimurium* | | 100 | 160 | | 236,956 | | 296 | 1 | Thanki et al 2019 | |
| VP4B | | *Vibrio harveyi* | | NA | NA | | 236,053 | | 212 | NA | NA | |
| vB_EamM_Joad | | *Erwinia amylovora* | | NA | NA | | 235,374 | | 245 | NA | Esplin et al 2017 | |
| 65 | | *Aeromonas salmonicida* | | NA | NA | | 235,229 | | 437 | 16 | Petrov et al 2010 | |
| vB_EamM_RisingSun | | *Erwinia amylovora* | | NA | NA | | 235,108 | | 243 | NA | Esplin et al 2017 | |
| vb_AbaM_ME3 | | *Acinetobacter baumanni* | | NA | NA | | 234,900 | | 326 | 4 | Buttimer et al 2016 | |
| ZPAH34 | | *Aeromonas hydrophila* | | 53.4±1.7 | 22.5±3 | | 234, 546 | | 234 | 2 |  | |
| Aeh1 | | *Aeromonas hydrophila* | | NA | NA | | 233,234 | | 352 | 27 | Chow and Rouf 1983 | |
| SPFM16 | | *Salmonella* *Typhimurium* | | 100 | 160 | | 233,195 | | 249 | 1 | Thanki et al 2019 | |
| S-SSM7 | | *Synechococcus* | | NA | NA | | 232,878 | | 319 | 5 | Sullivan et al 2010 | |
| Phage | Host | | | Virion size (nm) | | | Genome length (nt) | | No. of CDSs | No. of tRNA | | References |
|  |  | | | Head | Tail length | |  | |  |  | |  |
| Xoo-sp14 | | *Xanthomonas oryzae pv.oryzae* | | NA | NA | | 232,104 | | 251 | 0 | Nazir et al 2020 | |
| pVa-21 | | *Vibrio alginolyticus* | | 87 | 240 | | 231,998 | | 241 | 0 | Kim et al 2019 | |
| CC2 | | *Aeromonas hydrophila* | | NA | NA | | 231,743 | | 427 | 9 | Shen et al 2012 | |
| phiRSL1 | | *Ralstonia solanacearum* | | 150 | 138 | | 231,255 | | 343 | 3 | Yamada et al 2010 | |
| AS-yj | | *Aeromonas salmonicida* | | 120.1 | 130.1 | | 230,183 | | 418 | 9 | Ling Chen et al 2018 | |
| AS-sw | | *Aeromonas salmonicida* | | 102.5 | 107.2 | | 230,024 | | 414 | 9 | Ling Chen et al 2018 | |
| AS-zj | | *Aeromonas salmonicida* | | 101.5 | 108.2 | | 230,023 | | 412 | 8 | Ling Chen et al 2018 | |
| ΦMV-1 | | *Morganella morganii* | | 116 | 121 | | 230,000 | | NA | NA | Yamaki et al 2019 | |
| AS-szw | *Aeromonas salmonicida* | | | 115.9 | 123.2 | | 229,957 | | 409 | 9 | Ling Chen et al 2018 | |
| vB_EamM_Phobos | *Erwinia amylovora* | | | NA | NA | | 229,501 | | 247 | NA | Esplin et al 2017 | |
| ACG-2014f^c^ | *Synechococcus* | | | NA | NA | | 228,143 | | 292 | NA | Gregory et al 2016 | |
| vB_OspM_OC | *Ochrobactrum spp* | | | 97.6 | 102.9 | | 227,654 | | 414 | 24 | Decewicz et al 2020 | |
| FLC6 | *Burkholderia glumae* | | | 150 | 225 | | 227,105 | | 241 | 0 | Sasaki et al 2021 | |
| PTm5 | *Tenacibaculum maritimum* | | | NA | NA | | 226,876 | | 306 | NA | Kawato et al 2020 | |
| Phage | Host | | | Virion size (nm) | | | Genome length (nt) | | No. of CDSs | No. of tRNA | | References |
|  |  | | | Head | Tail length | |  | |  |  | |  |
| phiAS5 | *Aeromonas salmonicida* | | | 121×71 | 98 | | 225,268 | | 343 | 24 | Kim et al 2012 | |
| PTm1 | *Tenacibaculum maritimum* | | | 120 | 150 | | 224,680 | | 308 | NA | Kawato et al 2020 | |
| CR5 | *Cronobacter sakazakii* | | | NA | NA | | 223,989 | | 231 | NA | Lee et al 2016 | |
| vB_EamM_EarlPhillipIV | *Erwinia amylovora* | | | NA | NA | | 223,935 | | 241 | NA | Esplin et al 2017 | |
| RSL2 | *Ralstonia solanacearum* | | | NA | NA | | 223,932 | | 224 | NA | Bhunchoth et al 2016 | |
| Rogue | *Caulobacter crescentus* | | | 205×60 | 319 | | 223,720 | | 350 | 23 | Meczker et al 2014 | |
| RSF1 | *Ralstonia solanacearum* | | | NA | NA | | 222,888 | | 230 | NA | Bhunchoth et al 2016 | |
| PX29 | *Aeromonas salmonicida* | | | NA | NA | | 222,006 | | 330 | 25 | Petrov et al 2010 | |
| SP-15 | *Bacillus subtilis* | | | NA | NA | | 221,908 | | 317 | NA | Taylor and Thorne et al 1963 | |
| Karma | *Caulobacter crescentus* | | | 205×61 | 314 | | 221,828 | | 353 | 26 | Meczker et al 2014 | |
| PAU | *Sphingomonas Paucimobilis* | | | NA | NA | | 219,372 | | 295 | 7 | White and Suttle 2013 | |
| Swift | *Caulobacter crescentus* | | | 219×63 | 295 | | 219,216 | | 343 | 27 | Meczker et al 2014 | |
| 0305phi8-36 | *Bacillus thuringiensis* | | | 95 | 486 | | 218,948 | | 246 | 0 | Serwer et al 2007 | |
| Magneto | *Caulobacter crescentus* | | | 211×58 | 293 | | 218,929 | | 347 | 27 | Meczker et al 2014 | |
| Phage | Host | | | Virion size (nm) | | | Genome length (nt) | | No. of CDSs | No. of tRNA | | References |
|  |  | | | Head | Tail length | |  | |  |  | |  |
| PhiEaH1 | *Erwinia amylovora* | | | NA | NA | | 218,339 | | 241 | NA | Meczker et al 2014 | |
| S-CAM7 | *Synechococcus* | | | NA | NA | | 216,121 | | 266 | NA | NA | |
| phiCbK | *Caulobacter crescentus* | | | 205×56 | 300 | | 215,710 | | 338 | 26 | Meczker et al 2014 | |
| EL | *Pseudomonas aeruginosa* | | | 140 | 200 | | 211,215 | | 201 | NA | Hertveldt et al 2005 | |
| S-SKS1 | *Synechococcus* | | | NA | NA | | 208,007 | | 281 | 11 | NA | |
| phiN3 | *Sinorhizobium* | | | NA | NA | | NA | | 402 | 6 | NA | |

NA indicated the data is not available.

**
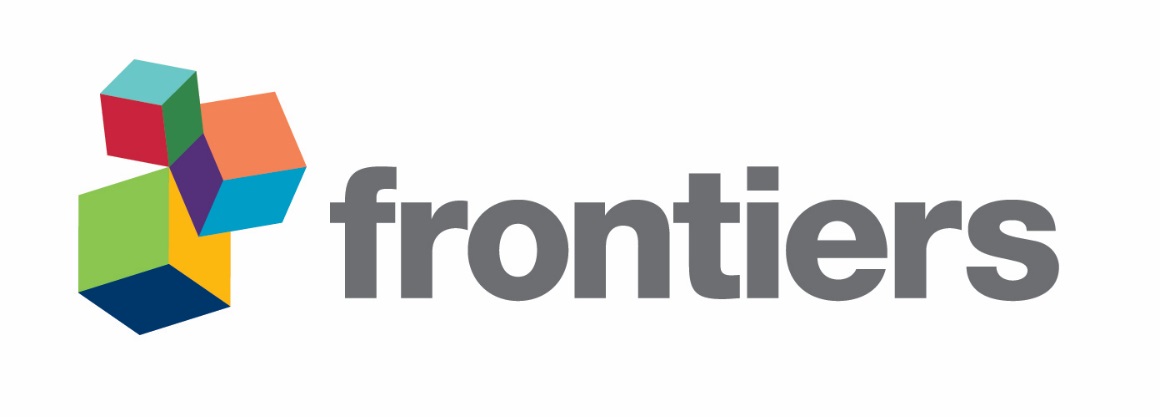
**
